# Relative sensory sparing in the diabetic foot implied through vibration testing

**DOI:** 10.3402/dfa.v4i0.21278

**Published:** 2013-09-16

**Authors:** Todd O'Brien, Joseph Karem

**Affiliations:** 1 Health Access Network, Lincoln, ME; 2 Independant Consultant

**Keywords:** diabetic foot, diabetes mellitus, diabetic polyneuropathy, diabetes-related complications, neurological examination, sural nerve

## Abstract

**Background:**

The dorsal aspect of the hallux is often cited as the anatomic location of choice for vibration testing in the feet of diabetic patients. To validate this preference, vibration tests were performed and compared at the hallux and 5th metatarsal head in diabetic patients with established neuropathy.

**Methods:**

Twenty-eight neuropathic, diabetic patients and 17 non-neuropathic, non-diabetic patients underwent timed vibration testing (TVT) with a novel 128 Hz electronic tuning fork (ETF) at the hallux and 5th metatarsal head.

**Results:**

TVT values in the feet of diabetic patients were found to be reduced at both locations compared to controls. Unexpectedly, these values were significantly lower at the hallux (P<0.001) compared to the 5th metatarsal head.

**Conclusion:**

This study confirms the hallux as the most appropriate location for vibration testing and implies relative sensory sparing at the 5th metatarsal head, a finding not previously reported in diabetic patients.

Diabetic peripheral neuropathy (DPN) has been established as an essential precursor leading to foot ulcers, infections, and lower extremity amputation (1). Effective clinical screening for DPN and implementation of preventative strategies designed to reduce complications leading to limb loss have been acknowledged as critical aspects in the care of diabetic patients (2, 3).

One of the most frequently used neurological screening tools for DPN is the 128 Hz tuning fork. The dorsal aspect of the distal phalanx of the hallux has long been cited as the anatomic location of choice for vibration testing with the tuning fork (4–8). Alternatively, some researchers have advocated vibration testing at other sites, including the fifth metatarsal head (9, 10). This study was undertaken to validate the choice of the hallux as the preferred site for vibration testing in the diabetic foot.

## Methods

To test this recommendation, tuning fork testing was performed at the hallux and 5th metatarsal head in diabetic patients with neuropathy (neuropathy diagnosis was established by conventional neurological testing). Standardization of testing between test subjects and anatomic sites was improved through the use of a novel 128 Hz electronic tuning fork (ETF) (11). This device electronically reproduces the same vibration output and decay rate as the traditional tuning fork. An integrated timer facilitates performance of timed vibration tests (TVTs), which have been shown to be a valid method of detecting neuropathy (12, 13). TVT values at the hallux and 5th metatarsal head were collected and analyzed.

Fifty-five patients were recruited on a rolling basis over a 6-month period for participation in the study at the Health Access Network, Lincoln, ME (Table 1). Criteria included being older than 18 years. Exclusion criteria included foot amputation, open foot ulcers, or foot infection. Diabetes diagnosis was determined by medical history. For the purposes of this study, patients were further subdivided into a diabetic, neuropathic group and a non-diabetic, non-neuropathic group. Comparison between these two groups was then made with regard to vibratory sensation. The study protocol was approved by the institutional review board administered by Portable Ethics IRB of Windham, ME. Written informed consent was obtained from all participants in this study.

**Table 1:** Patient demographics

Variable	Diabetic, neuropathic patients	Non-diabetic, non-neuropathic patients
N	28	17
Male (%)	16 (57.1)	3 (17.6)
AGE (years)		
Mean ± SD	64.6 ± 12.3	53.2 ± 20.1
Median	64.0	57.0
Range	32–88	20–83

To diagnose neuropathy, each test subject underwent testing with a 5.07/10 g Semmes-Weinstein monofilament test (SWMT) (Touch-Test; North Coast Medical, Morgan Hill, CA), biothesiometer (Biomedical Instruments, Newbury, OH), sharp/dull discrimination test, and ETF (O'Brien Medical, Orono, ME). An abnormal result from any one of the conventional tests (SWMT, biothesiometer, sharp/dull discrimination test) qualified a test subject as neuropathic. Each test was administered in a treatment room with an ambient temperature of 70–72°F. Test subjects had their socks removed for 5–10 min prior to testing.

## Specific testing protocols were as follows

*SWMT*: The accuracy of monofilaments was assessed daily with a digital scale prior to testing. Monofilaments registering beyond ±5% of the desired 10 g of pressure were not used. Standard technique (2) was used when applying the monofilaments to the plantar aspects of the 1st and 5th digits. Test subjects, with eyes closed, would verbally indicate perception of the monofilament touch by saying ‘yes’. Lack of an expected response at any location constituted an abnormal reading.

*Biothesiometer*: The biothesiometer was set at the 25-v level and applied to the dorsal aspect of the distal phalanx of the hallux and the dorsal aspect of the 5th metatarsal head. Test subjects would verbally indicate if they perceived vibrations with a ‘yes’ or ‘no’. Lack of patient perception at any location was recorded as an abnormal reading.

*Sharp/Dull Discrimination Test*: A sharply cut monofilament imparting 60 g, ±5% was applied for sharp touch. A monofilament terminating in a blunt polyisoprene tip imparting 225 g, ±5% was applied for dull touch. Standard technique (2) was used when applying the monofilaments to the plantar aspects of the 1st and 5th digits. Test subjects, with eyes closed, would indicate if they perceived the touch of the instrument as either ‘sharp’ or ‘dull’. Incorrect responses at any location constituted an abnormal reading.

**Fig. 1:**
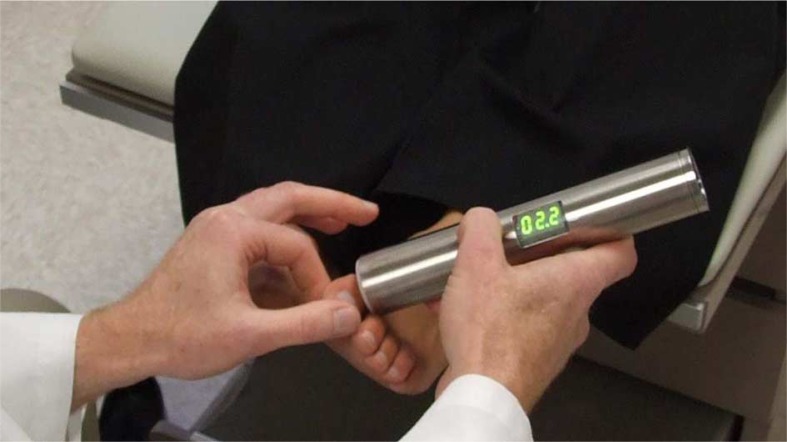
The ETF applied to the hallux.

*ETF*: The contact point of the ETF was applied to the dorsal aspect of the distal phalanx of the hallux and the dorsal aspect of the 5th metatarsal head (Fig. 1). The device was activated, simultaneously starting the vibrations and integrated timer. Test subjects would verbally indicate if they perceived vibrations with a ‘yes’ or ‘no’. Those indicating ‘no’ were recorded as 0 sec elapsed time. Those indicating ‘yes’ were asked to state when the vibrations subsided beyond their perception by saying ‘now’. At this point, the device was stopped and the elapsed time was recorded. In addition to neurological testing, patient demographic and diabetes history were also collected. A paired *t*-test was done on the data from the ETF to determine if there was any difference in sensitivity between the hallux and 5th metatarsal head in participating patient feet. No transformation of patient data was necessary to meet the assumptions of normality. Normality was assessed by examining skewness, kurtosis, and the Shapiro-Wilk W statistic.

## Results

TVT values from 45 of the 55 patients examined are presented in these results (Table 1). Twenty-eight met the criteria for inclusion in the diabetic, neuropathic group, and 17 met the criteria for inclusion in the non-diabetic, non-neuropathic group. Ten patients did not fall into either group so their TVT values were not evaluated.

Quantitative results of ETF testing showed that diabetic patients with neuropathy exhibited a substantial loss in vibratory sensation at both the hallux and 5th metatarsal head when compared to non-diabetic, non-neuropathic patients. Mean readings using the ETF showed that non-diabetic patients detected vibration an average of three times longer at the hallux and twice as long at the 5th metatarsal head compared to diabetic patients with neuropathy (Table 2). ETF readings also indicate a greater loss of sensitivity at the hallux compared to the 5th metatarsal head in the feet of diabetic patients with neuropathy (P = 0.001), and no difference between these sites in non-diabetic patients (P = 0.556). Overall, neuropathic diabetic patients perceived vibrations from the ETF 2.42 sec longer at the 5th metatarsal head compared to the hallux (Table 2).

**Table 2:** An ETF comparison of sensitivity at the hallux versus the 5th metatarsal head for diabetic patients with neuropathy and non-diabetic patients

Variable	Diabetic, neuropathic patients	Non-diabetic, non-neuropathic patients
N (feet examined)	54*	34
Hallux Mean ETF (sec)	3.57	11.76
5th MTh Mean ETF (sec)	5.99	12.19
Paired Test (hallux – 5th MTh)		
Mean difference (sec)	−2.42	−0.43
SD (sec)	5.17	4.18
P	0.001	0.556

* Individual feet were treated as independent, statistical elements for the purposes of this study. Data were erroneously not collected on 2 feet from two different patients. The resultant data set from the 28 diabetic, neuropathic patients therefore yielded only 54 feet.

## Discussion

In agreement with current clinical guidelines, the results from this study indicate that the dorsal aspect of the distal phalanx of the hallux is an appropriate location for vibration testing in diabetic patients. Unexpectedly, TVT values were found to be significantly longer at the 5th metatarsal head when compared to the hallux. This finding suggests relatively slower progression of DPN at the 5th metatarsal head. Supporting this conclusion are several studies comparing the electrodiagnostic sensitivity of the sural nerve (afferent to the 5th metatarsal head) versus the medial plantar nerve (afferent to the hallux).

In particular, Sylantiev et al. compared nerve conduction velocities at the sural and medial plantar nerves in patients with and without symptoms of distal symmetrical polyneuropathy (DSP) (14). It was found that the medial plantar nerve was a more sensitive choice for diagnosing DSP than the sural (90 vs. 55% sensitivity). It was noted that this finding was contrary to the traditional recommendation of using the sural nerve for electrodiagnostic testing of DSP. The authors concluded that the medial plantar nerve provided a higher diagnostic yield and advocated for preferential testing of this nerve. Similarly, others have independently confirmed that electrodiagnostic testing of the medial plantar nerve provides increased diagnostic sensitivity in diabetic patients when compared to the sural (15–18). Further research on this topic appears warranted given the widespread clinical reliance on electrodiagnostic testing of the sural nerve in a variety of neurological disorders.

One limitation of this study was the individual patient population derived from a single physician practice. Although statistically significant findings were clearly evident, a meta-analysis would serve to confirm our results. Additionally, clinical data collection was carried out by one author who was not blinded to the results of the conventional neurological screening tests (biothesiometer, SWMT and sharp/dull discrimination test) or the patient's diabetes status.

Another limiting factor was the omission of vibration testing at the plantar aspect of the hallux (i.e. the pulp of the toe). This site has also been advocated by researchers but never directly compared to the dorsal site (2, 19). Although vibrations transmitted through the hallux do stimulate dorsal and plantar mechanoreceptors, it is possible that the plantar site is a more sensitive location for identifying neuropathy. The choice of this alternative location is also supported by the recent focus on the medial plantar nerve in electrodiagnostic research. Inclusion of the plantar hallux site in this study could have provided guidance on this question.

The fact that a unique prototype was used in this study may also be a limiting factor. Although the ETF was designed to replicate the output of a traditional tuning fork, there could be subtle differences between the two. It is not clear if these differences are clinically significant. Additionally, the reproducible vibrations created by the device likely mitigated significant variation between tests.

In conclusion, evidence of relative sensory sparing in diabetic patients with DPN was found when comparing vibratory sensation at the hallux and 5th metatarsal head. This finding, implying more rapid progression of neuropathy at the hallux, should provide clinicians with a high level of confidence that this is the optimal location for vibration testing in the diabetic foot.
